# Acute Rheumatic Fever: A Review of Essential Cutaneous and Histological Findings

**DOI:** 10.7759/cureus.12577

**Published:** 2021-01-08

**Authors:** Matthew A Heard, Margaret C Green, Michael Royer

**Affiliations:** 1 College of Osteopathic Medicine, University of New England, Biddeford, USA; 2 Dermatology, Walter Reed National Military Medical Center, Bethesda, USA; 3 Dermatopathology, Joint Pathology Center, Silver Spring, USA

**Keywords:** acute rheumatic fever, group a streptococcus, dermatopathology, erythema marginatum, subcutaneous nodule, migratory arthritis

## Abstract

Acute rheumatic fever (ARF) is an autoimmune response that may occur after infection with group A *Streptococcus*. Clinical manifestations are protean, making the syndrome difficult to recognize in the 21st century. Secondary prophylaxis with benzathine penicillin is given for 10 years after an episode of ARF to prevent recurrence and reduce the risk of rheumatic heart disease. This case highlights the importance of providing a detailed clinical history to the dermatopathologist when considering ARF in the differential diagnosis.

## Introduction

Skin manifestations of acute rheumatic fever (ARF) include erythema marginatum and subcutaneous nodules [1]. Both are major manifestations of the Jones criteria but are rarely seen or biopsied in the United States due to a low disease incidence in most of the country [2]. We present the case of an 18-year-old male admitted for three days of fever, incapacitating migratory arthritis, and subcutaneous nodules on the extremities, chest, and back one week after developing a sore throat and diarrhea. Biopsy of a subcutaneous nodule showed superficial and deep dermal lymphohistiocytic inflammation, extending into the subcutis, poorly formed granulomas with central necrosis, acute inflammation, and leukocytoclasis. Other laboratory abnormalities included an elevated antistreptolysin-O (ASO) titer, erythrocyte sedimentation rate (ESR) and C-reactive protein (CRP). This constellation of findings was consistent with two major (subcutaneous nodules and arthritis) and two minor (fever and elevated CRP) Jones criteria [1]. Treatment of ARF includes appropriate antibiotic coverage and systemic anti-inflammatory medications.

## Case presentation

**Figure 1:**
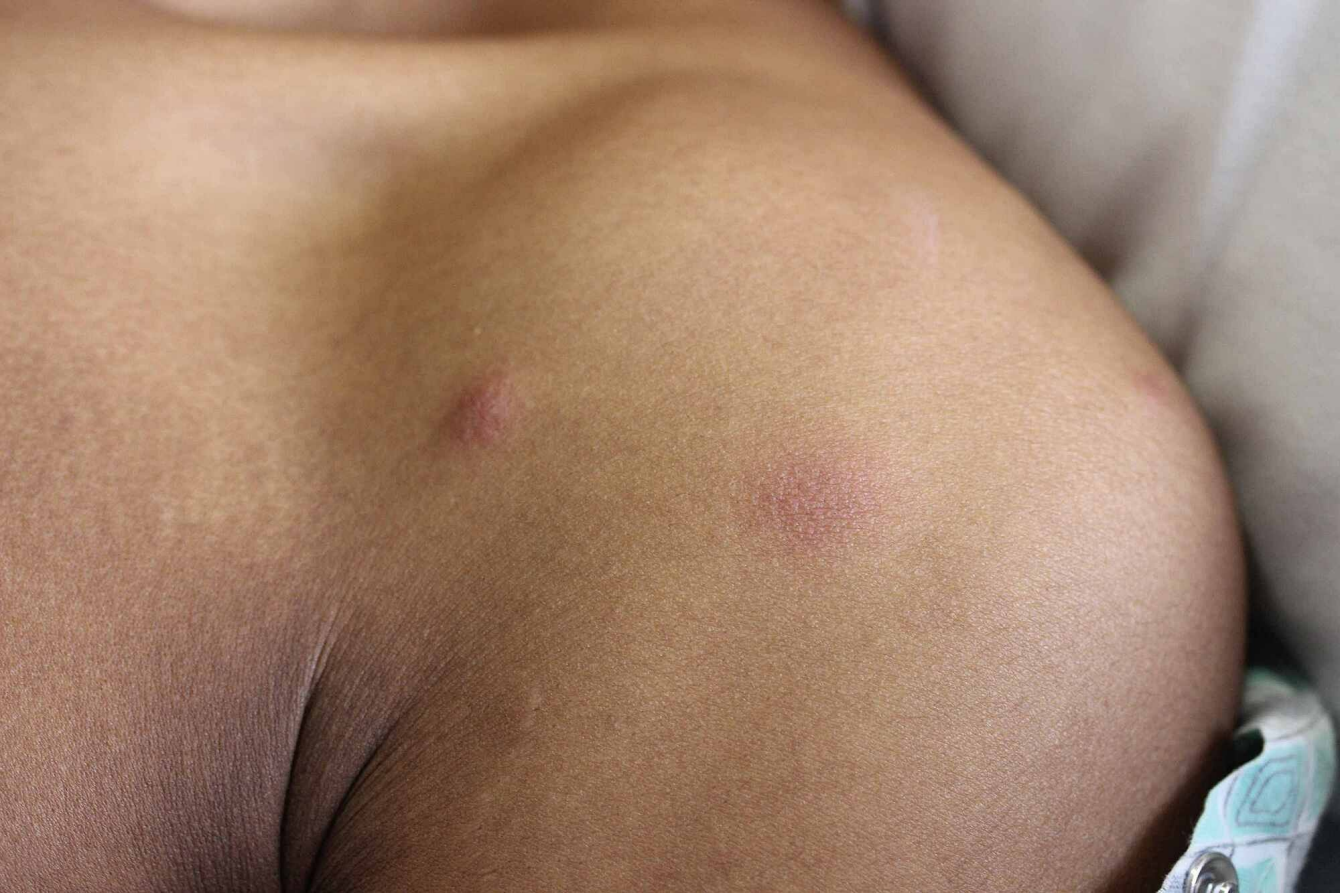
Subcutaneous nodules on the left anterior shoulder and chest with faint overlying erythema.

An 18-year-old African American male developed fever, nausea, diarrhea, sore throat, and swollen lymph nodes in the neck and inguinal creases. Three days later, his left ankle became swollen and painful, followed by right ankle involvement. He then developed tender, warm nodules on his arms, chest, back, and lower legs (Figure 1).

**Figure 2:**
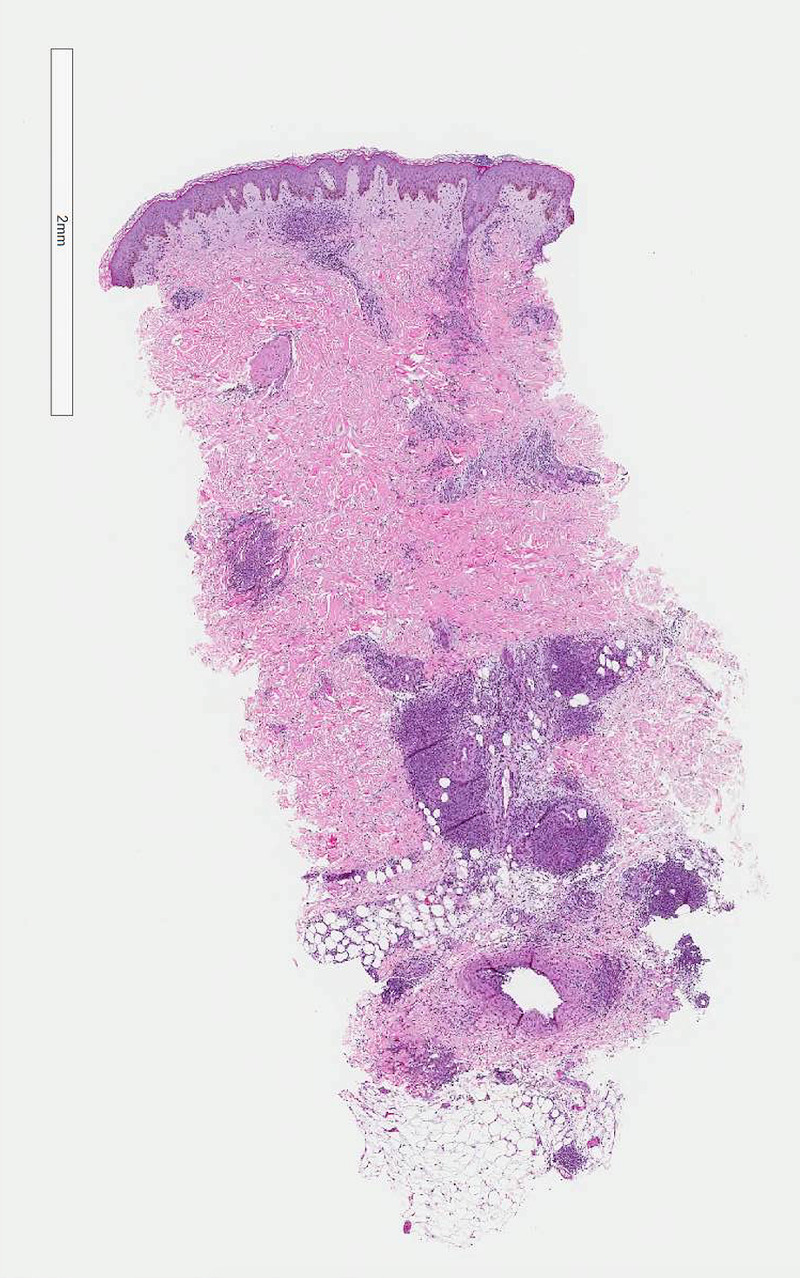
Superficial and deep perivascular lymphohistiocytic inflammation, extending into the subcutis (hematoxylin & eosin, 2× magnification).

**Figure 3:**
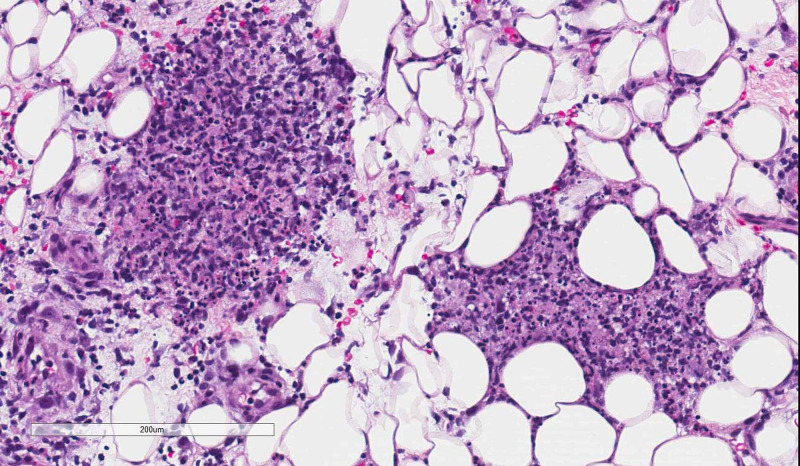
Subcutaneous mixed acute and chronic inflammation (hematoxylin & eosin, 10× magnification).

**Figure 4:**
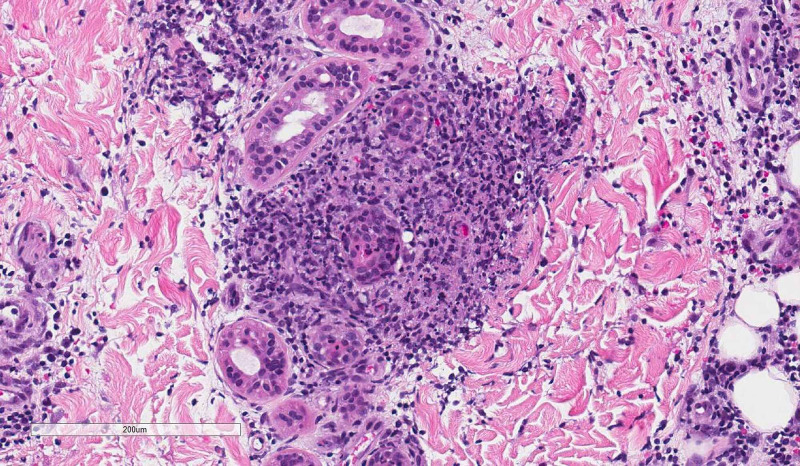
Poorly formed peri-eccrine granuloma with peripheral leukocytoclasis (hematoxylin & eosin, 10× magnification).

A week after his symptoms began, he presented to the emergency department and was admitted. Results pertinent to the case included a negative rapid *Streptococcus* A test, throat culture, and synovial fluid culture. The patient’s ESR was 41 mm/h and CRP was 190.00 mg/L. A skin biopsy of a representative subcutaneous nodule was performed that demonstrated superficial and deep dermal lymphohistiocytic inflammation, extending into the subcutis (Figures 2, 3). There were poorly formed granulomas with central coagulative necrosis and peripheral acute inflammation with conspicuous leukocytoclasis (Figure 4). Special stains for infectious organisms (Periodic acid-Schiff, Grocott’s methenamine silver, Ziehl-Neelsen, acid-fast bacilli, and tissue gram stain) were negative. Due to a lack of supportive clinical history, these microscopic findings were considered non-specific.

Despite negative testing, there was initial concern for disseminated gonococcemia or reactive arthritis, and he was treated with azithromycin and ceftriaxone until an elevated ASO titer (473.0 IU/mL) was reported. He then received treatment with a single dose of intramuscular benzathine penicillin and systemic corticosteroids with improvement of symptoms over the course of a week. His transthoracic echocardiogram was normal. He was advised to receive benzathine penicillin every four weeks and repeat echocardiography after one year. Unfortunately, the patient received three monthly doses before he was lost to follow-up.

## Discussion

Background/Epidemiology

Although ARF is now an uncommon disorder in the United States, it continues to cause significant morbidity and mortality globally. The number of new cases per year is approximately 471,000 with over 300,000 deaths from either ARF or rheumatic heart disease (RHD), one of the chronic sequelae that is estimated to occur in 60% of those affected by ARF [2]. The number of people living with RHD is estimated to be 33 million [3]. ARF most often affects children aged 5 to 14 years, and has been linked to low socioeconomic status with overcrowding, rural locations, and poor access to medical care [1]. There is evidence for genetic susceptibility for developing ARF and RHD, which will continue to change with evolution of both humans and* Streptococcus pyogenes *as well as with globalization [4].

Clinical features and immunology

ARF is an autoimmune response to a preceding infection with group A *Streptococcus* (GAS) (*S. pyogenes*), and has been reported after both pharyngitis and impetigo [5]. The immune response to GAS infection includes activation of B and T lymphocytes by various antigen-presenting cells after they interact with the bacteria. These lymphocytes subsequently mount an immune response against bacterial epitopes. Autoimmunity is thought to develop through molecular mimicry between bacterial-specific epitopes and antibodies and similarly structured host tissues. Immune complexes form between these antibodies and the host’s tissue, with subsequent activation of an inflammatory response and tissue damage [1]. The organ systems most commonly affected in ARF are the heart, joints, skin, and central nervous system. The clinical manifestations of ARF are summarized by the Jones criteria (Table 1). They are divided into major and minor criteria and between low- and moderate-to-high-risk populations [6]. Low-risk populations are defined based on ARF incidence of ≤2 per 100,000 school-aged children or all-age RHD prevalence of ≤1 per 1,000 people per year [6].

**Table 1:** Revised Jones criteria. Initial diagnosis requires two major criteria or one major and two minor criteria. Diagnosis of recurrent ARF requires two major criteria, one major and two minor criteria or three minor criteria (Gewits et al, 2015). ARF, acute rheumatic fever; CRP, C-reactive protein; ESR, erythrocyte sedimentation rate

Revised Jones criteria	
Major criteria	
Low-risk population	Moderate-to-high-risk population
Carditis (detected with echocardiography), polyarthritis, chorea erythema marginatum, subcutaneous nodules	Carditis (detected with echocardiography), mono- or polyarthritis, polyarthralgia chorea, erythema marginatum, subcutaneous nodules
Minor Criteria	
Low-risk population	Moderate-to-high-risk population
Polyarthralgia, fever of ≥38.5°C, ESR of ≥60 mm in the first hour and/or CRP of ≥3.0 mg/dL, prolonged PR interval	Monoarthralgia, fever of ≥38°C, ESR of ≥30 mm in the first hour and/or CRP of ≥3.0 mg/dL, prolonged PR interval

Although the cutaneous manifestations of ARF are within the major criteria, erythema marginatum and subcutaneous nodules are reported to occur in less than 5% of the cases [7]. The clinical presentation of the subcutaneous nodules has been described as varying from a few millimeters to a few centimeters in size and favoring periarticular locations and overlying bony prominences. They typically occur in clusters, with duration ranging from a few days to several months. These subcutaneous nodules are important to recognize because they are a harbinger for involvement of cardiac valves and worsened severity of RHD [7,8].

Histologic features

The first histologic descriptions of subcutaneous nodules of ARF were published in the early 1880s, and the term “*rheumatismus nodosus*” was first used in 1885 [9]. The histologic appearance varies whether an early or an established nodule is biopsied. Features in early descriptions include increased numbers of dilated blood vessels with peripherally thickened walls, fibrinoid necrosis, and fibrosis with attachment to tendons [9]. They are histologically similar to nodules seen in rheumatoid arthritis with central fibrinoid necrosis, surrounded by histiocytes and perivascular lymphocytes and neutrophils [8].

Treatment

In GAS infections, use of appropriate antibiotics in confirmed cases is essential for the primary prevention of ARF. Acceptable forms of treatment include intramuscular benzathine penicillin, oral penicillin twice a day for 10 days, and oral amoxicillin once daily for 10 days [1]. Once a patient has developed ARF, benzathine penicillin is used for prevention of recurrent ARF and progression of RHD. According to the World Health Organization, this treatment should be administered every three to four weeks and continued for a minimum of 10 years in patients with carditis, but it may be continued for a longer duration in severe cases [7]. Fortunately, the cutaneous manifestations of ARF are typically asymptomatic and resolve gradually without treatment.

Overview

This case occurred in the United States military health system, which has a diverse and relatively young population. Military physicians are sometimes called upon to treat foreign populations in the setting of deployment or humanitarian outreach. It is imperative to consider and recognize well-described manifestations of streptococcal infections, despite their low prevalence in most of the United States. This report highlights how essential it is to provide the dermatopathologist with detailed clinical history and, in some cases, patient photographs for appropriate diagnosis. Clinically, this was a challenging case because the initial testing for GAS was negative. The diagnosis was not suspected, even by infectious disease and dermatology physicians, as the Jones criteria would not be applicable without evidence of preceding streptococcal infection.

There are multiple factors that should be considered when deciding on the appropriate duration of secondary prophylaxis with benzathine penicillin. These factors include likelihood of another exposure to GAS, the age of the patient, and whether the patient had carditis during the ARF episode or at follow-up [2]. Typically, secondary prophylaxis is continued for a minimum of 10 years after the ARF episode [2]. Because the goal is to prevent recurrent ARF and recurrence is less common after the ages of 25 to 30, stopping prophylaxis in this patient after seven years (i.e., when he is 25 years old) could be considered [2]. Presence of RHD would support secondary prophylaxis for a longer duration, until age 30 to 35 for moderate RHD and until age 40 for severe RHD [2].

## Conclusions

This report describes a case of ARF that was not initially considered as a potential diagnosis in an otherwise healthy young male. It highlights the epidemiology, clinical and histologic features, and treatment of ARF. It also reinforces the importance of providing a detailed history to the dermatopathologist when submitting specimens and to consider the possibility of a rare disease in a population with low prevalence.
